# Targeting mitochondrial reactive oxygen species-mediated oxidative stress attenuates nicotine-induced cardiac remodeling and dysfunction

**DOI:** 10.1038/s41598-021-93234-4

**Published:** 2021-07-05

**Authors:** Anand Ramalingam, Siti Balkis Budin, Norsyahida Mohd Fauzi, Rebecca H. Ritchie, Satirah Zainalabidin

**Affiliations:** 1 Program of Biomedical Science, Centre of Toxicology and Health Risk Studies (CORE), Faculty of Health Sciences, Universiti Kebangsaan Malaysia, Kuala Lumpur, Malaysia; 2 Program of Biomedical Science, Centre of Diagnostic, Therapeutic and Investigative Studies, Faculty of Health Sciences, Universiti Kebangsaan Malaysia, Kuala Lumpur, Malaysia; 3 Drug and Herbal Research Centre, Faculty of Pharmacy, Universiti Kebangsaan Malaysia, Jalan Raja Muda Abdul Aziz, Kuala Lumpur, Malaysia; 4 Heart Failure Pharmacology, Baker Heart and Diabetes Institute, Melbourne, VIC Australia; 5 Drug Discovery Biology, Monash Institute of Pharmaceutical Sciences, Monash University, Parkville, VIC Australia

**Keywords:** Pharmacology, Drug discovery, Cardiology

## Abstract

Long-term nicotine intake is associated with an increased risk of myocardial damage and dysfunction. However, it remains unclear whether targeting mitochondrial reactive oxygen species (ROS) prevents nicotine-induced cardiac remodeling and dysfunction. This study investigated the effects of mitoTEMPO (a mitochondria-targeted antioxidant), and resveratrol (a sirtuin activator) , on nicotine-induced cardiac remodeling and dysfunction. Sprague–Dawley rats were administered 0.6 mg/kg nicotine daily with 0.7 mg/kg mitoTEMPO, 8 mg/kg resveratrol, or vehicle alone for 28 days. At the end of the study, rat hearts were collected to analyze the cardiac structure, mitochondrial ROS level, oxidative stress, and inflammation markers. A subset of rat hearts was perfused ex vivo to determine the cardiac function and myocardial susceptibility to ischemia–reperfusion injury. Nicotine administration significantly augmented mitochondrial ROS level, cardiomyocyte hypertrophy, fibrosis, and inflammation in rat hearts. Nicotine administration also induced left ventricular dysfunction, which was worsened by ischemia–reperfusion in isolated rat hearts. MitoTEMPO and resveratrol both significantly attenuated the adverse cardiac remodeling induced by nicotine, as well as the aggravation of postischemic ventricular dysfunction. Findings from this study show that targeting mitochondrial ROS with mitoTEMPO or resveratrol partially attenuates nicotine-induced cardiac remodeling and dysfunction.

## Introduction

Cardiovascular disease remains the primary cause of death and disability in Malaysia and most developing and developed countries worldwide (including Malaysia), despite improvements in the healthcare sectors^1,2^. In 2017, cardiovascular disease alone accounted for ~ 17.8 million deaths worldwide, and this number is projected to increase by 25% in the next 10 years unless adequate measures are implemented to promote control of risk factors that include smoking, sedentary lifestyle, and unhealthy diet^1,2^. Among these risk factors, smoking, either active or passive, is one of the most important modifiable risk factors for cardiovascular disease globally.

Nicotine in tobacco smoke is frequently suggested to mediate the smoking-driven risk of cardiovascular diseases. Long-term exposure to nicotine has been associated with an increased risk of hypertension and atrial fibrillation^3,4^. A study conducted in rats has also shown that prolonged administration of nicotine (21–28 days) increases systemic blood pressure and heart rate^5^, accelerates cardiomyocyte inflammation and apoptosis^6^, promotes atherosclerosis^7^, dysregulates blood lipid profile^7^, and causes vascular endothelial dysfunction^5,8^. Our recent studies also showed that nicotine administration for 28 days causes cardiac structural remodeling such as hypertrophy and fibrosis, which render the hearts more susceptible to acute insults such as ischemia–reperfusion injury^9,10^. We also reported that hearts isolated from nicotine-administered rats exhibited worsened left ventricle (LV) function recovery^9,10^.

Oxidative stress is a critical mechanism underlying nicotine-induced cardiovascular diseases^8,10,11^. Several studies have shown that nicotine administration increases NADPH oxidase and mitochondria-driven reactive oxygen species (ROS) production in cardiomyocytes^9,10^. Increased lipid peroxidative damage and protein oxidation were evident in rat hearts after 21–28 days of nicotine administration^11^. Vitamin E and metallothionein both mitigated nicotine-induced oxidative stress, cardiomyocyte injury, and cardiovascular remodeling in animal models^12,13^. While these studies support a role for oxidative stress in nicotine-induced cardiovascular diseases, it is currently unclear how a more targeted approach that addresses the source of ROS, such as mitochondria, may impact nicotine-induced cardiovascular diseases. Although we have previously shown that mitochondrial ROS mediates the nicotine-induced increase in mitochondrial permeability transition pore opening, which is a critical determinant of myocardial ischemia–reperfusion injury^10^, it remains unclear whether targeting mitochondrial ROS emission would prevent nicotine-induced cardiac remodeling and dysfunction in vivo. We hypothesized that targeting mitochondrial ROS-driven oxidative stress may be more effective in preventing nicotine-induced cardiovascular diseases.

MitoTEMPO is a synthetic mitochondria-targeted SOD mimetic made from a combination of lipophilic cation triphenylphosphonium (TPP^+^) and the piperidine nitroxide (TEMPO) that scavenges superoxide and alkyl radicals^14^. TPP^+^ is highly membrane-permeable and allows mitoTEMPO to accumulate several hundred folds within the mitochondria and selectively target ROS within the mitochondria matrix. Several lines of evidence have now suggested that mitoTEMPO effectively attenuates cardiovascular disease such as diabetic cardiomyopathy by regulating mitochondrial ROS production^15,16^. MitoTEMPO also prevented heart failure and eliminated sudden cardiac death by decreasing ventricular arrhythmias and proteome remodeling^17^. Several studies also showed that mitoTEMPO derivatives protected against myocardial ischemia–reperfusion injury in aged hearts by attenuating ROS-driven mitochondrial permeability transition^18,19^.

Resveratrol (3,5,4-trihydroxystilbene) is a naturally occurring stilbene commonly found in red wine, grapes, blueberries, nuts, and cranberries. Many studies have established that resveratrol is cardioprotective in preclinical models and humans^20–22^. Resveratrol has been reported as a promising compound against myocardial ischemia–reperfusion injury, myocarditis, cardiac hypertrophy, and heart failure by suppressing oxidative stress, inflammation, pathological hypertrophic signaling, and apoptosis, as well as eliciting vasorelaxation and improving LV contractile function^20,23^. It is also well-documented that resveratrol is a sirtuin activator capable of potentiating sirtuins' deacetylase activity such as SIRT1 and SIRT3 and improving the antioxidant activities of sirtuin-regulated endogenous antioxidants such as superoxide dismutase 2 (SOD2) in mitochondria^23^. Resveratrol has also been shown to effectively mitigate oxidative and nitrosative stress by scavenging ROS such as superoxide and peroxynitrite^24^.

Overall, this study aimed to investigate the impact of targeting mitochondrial ROS-driven oxidative stress via either mitoTEMPO or resveratrol administration on nicotine-induced cardiac remodeling and dysfunction in rats. We specifically examined whether mitoTEMPO and resveratrol prevent nicotine-induced oxidative stress, inflammation, cardiac remodeling, and cardiac dysfunction. We also determined the impact of mitoTEMPO and resveratrol on nicotine-associated aggravation of the myocardial ischemia–reperfusion injury reported in our previous study^9,10^.

## Materials and methods

### Chemicals

Nicotine and resveratrol were purchased from Tokyo Chemical Industry (Tokyo, Japan), whereas mitoTEMPO was purchased from Sigma-Aldrich (St Louis, MO, United States). All other reagents used in this study were of laboratory analysis grade unless otherwise stated.

### Ethical statement

All experiments are reported in accordance with the Animal Research: Reporting In Vivo Experiments (ARRIVE) guidelines. All procedures in this study were approved by the UKM Animal Ethics Committee (UKMAEC) (FSK/BIOMED/2012/SATIRAH/12-DEC./486-DEC.-2012-DEC.2014).

### Animals

Male Sprague–Dawley rats (5–6 weeks old, 180–230 g) were purchased from Synertec Enterprise (Kuala Lumpur, Malaysia). They were housed under standard laboratory conditions in the Universiti Kebangsaan Malaysia (UKM) Kuala Lumpur Campus Animal Facility. Standard rodent pellet and tap water were provided ad libitum.

### Study design

After 1-week acclimatization, rats were randomly allocated into four groups: control, nicotine-alone (NIC), and nicotine plus mitoTEMPO (NIC + MT), and nicotine plus resveratrol (NIC + R). All rats (except those from the vehicle control group) were given 0.6 mg/kg nicotine dissolved in normal saline via intraperitoneal injection for 28 consecutive days as previously described^9,10^. All rats allocated to the NIC + MT group received 0.7 m/kg mitoTEMPO in DMSO vehicle intraperitoneally concomitant with nicotine for 28 days^25^. Resveratrol was also dissolved in DMSO and administered to rats allocated to the NIC + R group for 28 days intraperitoneally^26^. Vehicle control rats were given 0.25 mL saline and 0.25 mL DMSO vehicle injections throughout the study. DMSO vehicle (5%v/v) was prepared using sterile distilled water.

Throughout this study, in vivo measurements of systolic blood pressure (SBP) and heart rate were obtained at weekly intervals via the non-invasive tail-cuff method (CODA non-invasive blood pressure system, Kent Scientific, USA) as previously described^9^. All rats were habituated to the CODA system in a designated quiet room (27 ± 2 °C) for at least three consecutive days before acquiring baseline measurements.

At the end of this study, blood was collected from each animal via orbital sinus bleeding to assess plasma cotinine using an ELISA kit from Elabscience Biotechnology (Wuhan, China). Hearts collected from a subset of rats (n = 7–8 per group; n = 7 for control, n = 8 for NIC, n = 7 for NIC + MT, and n = 7 for NIC + R) were used for the analyses of heart structure, gene expression, mitochondrial ROS level, immunohistochemistry and antioxidant enzyme activity. Another subset of rats (n = 6–8/group; n = 6 for control, n = 7 for NIC, n = 8 for NIC + MT, and n = 7 for NIC + R) was used for the preparation of Langendorff heart to study changes in cardiac function and myocardial susceptibility to ischemia–reperfusion injury.

### Analysis of mitochondrial ROS production

Rat heart mitochondria were isolated from LV tissues as previously described^27^. Isolated mitochondria were resuspended in buffer containing 250 mM sucrose, 20 mM Tris–HCl, and 40 mM KCl^9,10^. Then, 200 µg of isolated mitochondria were loaded with 5 µM membrane-permeable fluorescent probe, MitoSOX Red (Invitrogen, USA), in the dark for 15 min at 37 °C^28^ in the presence of 5 mM glutamate and 5 mM malate as complex I respiratory substrates. Fluorescence intensity was measured using Varioskan multimode plate reader (Thermo Scientific, USA) at 510 nm excitation and 580 nm emission wavelengths and was expressed relative to the vehicle control group.

### Antioxidant assays

LV tissues were homogenized in ice-cold Tris–HCl buffer, and the resulting supernatant was used to detect superoxide dismutase (SOD2) activity as previously described^29^. GSH:GSSG ratio was determined using modified Ellman assay as described previously^30^. Total GSH (GSH + GSSG) level was first determined after incubation with NADPH and glutathione reductase via 5,5-dithio-bis-(2-nitrobenzoic acid) colorimetric reaction. GSSG level was then determined in tissue homogenate after derivatization with 2-vinylpyridine using the same method. GSH level was then calculated by subtracting GSSG value from GSH + GSSG value. Results were expressed as per mg protein content in the tissue homogenate pre-determined using Bradford protein assay^31^.

### Histology and immunohistochemistry

Formalin-fixed paraffin-embedded LV tissue sections (5 µm) were stained with hematoxylin and eosin (H&E), for measurement of cardiomyocyte size or picrosirius red, for measurement of collagen density in bright field microscopic images respectively^9^. For H&E staining, the cross-sectional area of ~ 100 cardiomyocytes per animal was quantified using Image J software from 10 independent bright-field images acquired under 40× magnification. Collagen density was also quantified using Image J software with macro from 10 separate bright-field images acquired under 10X magnification. LV sections (5 µm) were also used for immunohistochemical detection of oxidative stress marker, 3-nitrotyrosine, as previously described^9^. Bright-field microscopic images were acquired under 40× magnification using an Olympus microscope, and ten independent images per animal were analyzed using Image J software version 1.51 (NIH, USA). https://imagej.nih.gov/ij/download.html.

### Gene expression analysis

Total RNA from LV tissues was extracted using QIAzol lysis reagent (QIAGEN, Germany) and was reverse-transcribed as previously described^9^. Expression of SIRT1, SIRT3, SOD2, pro-hypertrophic genes (atrial natriuretic peptide, ANP, and B-type natriuretic peptide, BNP), fibrotic genes (transforming growth factor β1, TGFβ1, and fibronectin, Fn1), inflammation-related genes (tumor necrosis factor α, TNFα, interleukin 6, IL-6, interleukin 10, IL-10, annexin A1, ANXA1 and formyl peptide receptor 2, FPR2), as well as NOX2 subunit of NADPH oxidase (Nox2) were determined using the QuantiNova SYBR Green PCR Kit (QIAGEN, Germany). Quantitative analysis of gene expression was performed using Applied Biosystems Prism 7700 Sequence Detection Software, using the primer sequences generated from the GenBank (Supplementary Table). Comparative 2^−∆∆Ct^ method to used detect fold changes relative to the vehicle control group with ribosomal 18S as the housekeeping gene^9^.

### Isolated Langendorff-perfused heart preparation

For Langendorff heart studies, rats from each group were given sodium heparin (500 U/kg) as an anticoagulant and urethane (1.2 g/kg) for anesthesia via intraperitoneal injections^9^. Hearts were rapidly excised and subjected to retrograde perfusion via the aortic cannula under the constant pressure of ~ 60–70 mmHg, with Krebs–Henseleit buffer (containing 118 mM NaCl, 25 mM NaHCO_3_, 4.7 mM KCl, 1.2 mM KH_2_PO_4_, 1.2 mM MgSO_4_, 1.25 mM CaCl_2_, 11 mM glucose; 95% O_2_/5% CO_2_ maintained at 37 °C). Changes in coronary flow (CF) and perfusion pressure were monitored continuously using flow and pressure transducers. A water-filled latex balloon was inserted into the LV to monitor changes in heart rate and intraventricular pressure derivatives such as left ventricular systolic pressure (LVSP), left ventricular end-diastolic pressure (LVEDP), the maximum velocity of contraction (LV + dP/dt) as well as the maximum velocity of relaxation (LV − dP/dt). Changes in left ventricular developed pressure (LVDP) were calculated as the difference between LVEDP and LVSP. All data were acquired using LabChart Pro version 7.0 acquisition software (AD Instruments, Australia). The latex balloon was filled with ~ 150 µL water for all rats, and the balloon was adjusted carefully to achieve a baseline LVEDP of 0–5 mmHg. We have presented the plots for all markers except for LVEDP in percent relative to the pre-ischemic baseline to demonstrate % recovery of LV function and coronary flow. LVEDP was adjusted to a baseline of 0–5 mmHg to achieve isovolumic Langendorff setup, and increment in LVEDP is reported as absolute change because LVEDP increment (> 100% of baseline) is an indicator of diastolic dysfunction.

Rat hearts were equilibrated for 20 min with a steady-flow, and hearts exhibiting poor function (e.g., heart rate < 100 beats/min and LV + dP/dt < 1500 mmHg/sec) during equilibration were excluded from the study^9^. After equilibration, each heart was subjected to 20 min of global ischemia and 60 min of reperfusion^9^. The heart was immersed in a water-jacketed glass chamber throughout the procedure to maintain heart temperature at 37 °C. Coronary effluent was collected by collecting drips of the buffer using a cryovial placed directly under the apex at 10-min intervals throughout reperfusion for measurement of cardiac injury markers: cardiac troponin T (cTnT) and lactate dehydrogenase (LDH) using ELISA kits (Elabscience Biotechnology, Wuhan, China) and spectrophotometric assay^32^. Coronary effluent samples were kept frozen at − 80 °C immediately after the collection.

### Statistical analysis

All data are presented as mean ± standard error of the mean (SEM). One-way or two-way analysis of variance (ANOVA) followed by Tukey post hoc test was used to analyze differences between groups unless otherwise indicated. Statistical significance was considered at *p* < 0.05.

## Results

### Systematic characteristics

Table 1 shows the end-point analysis of body weight, organ weight, and plasma cotinine in all experimental groups. In this study, nicotine administration significantly increased heart weight, LV weight, the ratio of heart weight-to-tibia length (HW:TL), as well as, ratio of LV weight-to-tibia length (LV:TL) relative to the vehicle-administered controls after 28 days (all *p* < 0.05; Table 1). However, mitoTEMPO and resveratrol significantly attenuated nicotine-induced increases in heart weight, LV weight, HW:TL, and LV:TL (Table 1). Analysis of plasma cotinine also revealed comparable nicotine intake levels in NIC, NIC + MT, and NIC + R groups after 28 days of administration (Table 1).

**Table 1:** End-point analysis of body weight, organ weight, and plasma cotinine in rats.

Parameters	Vehicle (*n* = 13)	NIC (*n* = 15)	NIC + MT (*n* = 15)	NIC + R (*n* = 14)
Body weight (g)	287.2 ± 17.8	289.6 ± 12.1	280.6 ± 16.5	281.8 ± 20.5
Heart weight (mg)	1021.2 ± 33.6	1264.5 ± 59.6*	1022.8 ± 54.5^#^	1012.6 ± 53.2^#^
LV weight (mg)	501.4 ± 23.6	622.6 ± 31.8*	500.8 ± 34.2^#^	508.4 ± 31.6^#^
Tibia length (mm)	39.5 ± 4.2	40.6 ± 4.8	39.4 ± 7.8	42.5 ± 5.5
HW:TL (mg/mm)	20.5 ± 0.5	27.2 ± 1.8*	20.3 ± 1.7^#^	19.8 ± 2.1^#^
LV:TL (mg/mm)	10.2 ± 1.3	17.2 ± 1.3*	10.4 ± 1.2^#^	11.2 ± 0.9^#^
Cotinine (ng/mL)	-	171.8 ± 21.5	165.1 ± 25.9	170.6 ± 29.6

All values are given as mean ± SEM for *n* = 13–15/group.

*HW* heart weight, *LV* left ventricle, *MT, NIC* nicotine, *NIC + MT* nicotine + mitoTEMPO, *NIC + R* nicotine + resveratrol, *TL* tibia length.

*p < 0.05 versus vehicle group.

^#^*p* < 0.05 versus NIC group using one-way ANOVA with Tukey post hoc test.

**Figure 1:**
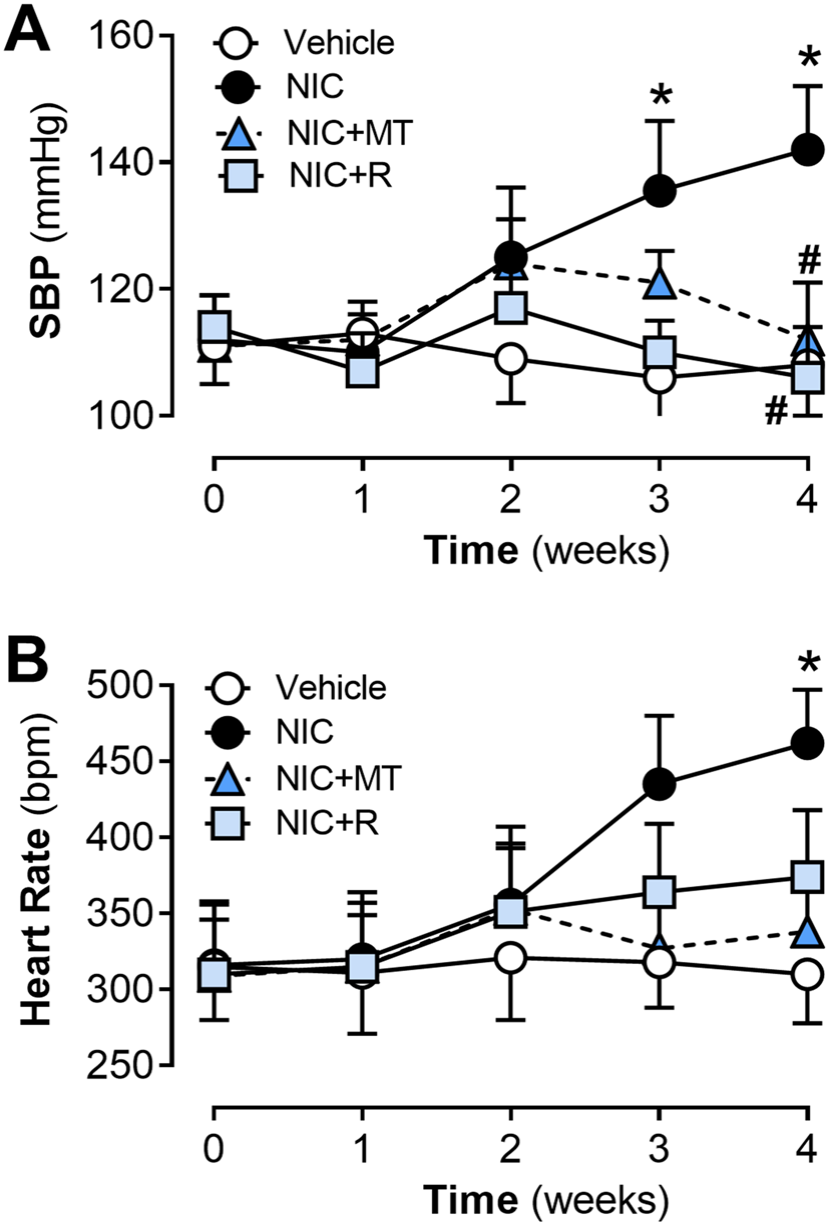
Time course changes in (**A**) SBP and (**B**) heart rate in rats from the vehicle, NIC, NIC + MT, and NIC + R groups over 28-days of treatment. All values are expressed as mean ± SEM for n = 13–15/group; *p < 0.05 versus vehicle group and #*p* < 0.05 versus NIC group using two-way ANOVA with repeated measurement.

On non-invasive tail-cuff blood pressure measurement, nicotine administration also increased systolic blood pressure (SBP) and heart rate in rats at 4 weeks post-administration, as compared to the vehicle controls (*p* < 0.05 for both; Fig. 1A, B). However, both mitoTEMPO and resveratrol significantly abolished the nicotine-induced increase in the end-point SBP (*p* < 0.05; Fig. 1A). Neither mitoTEMPO nor resveratrol had a significant impact on the heart rate of nicotine-administered rats (Fig. 1B).

### MitoTEMPO and resveratrol blunted nicotine-induced mitochondrial ROS emission and oxidative stress in rat hearts

**Figure 2:**
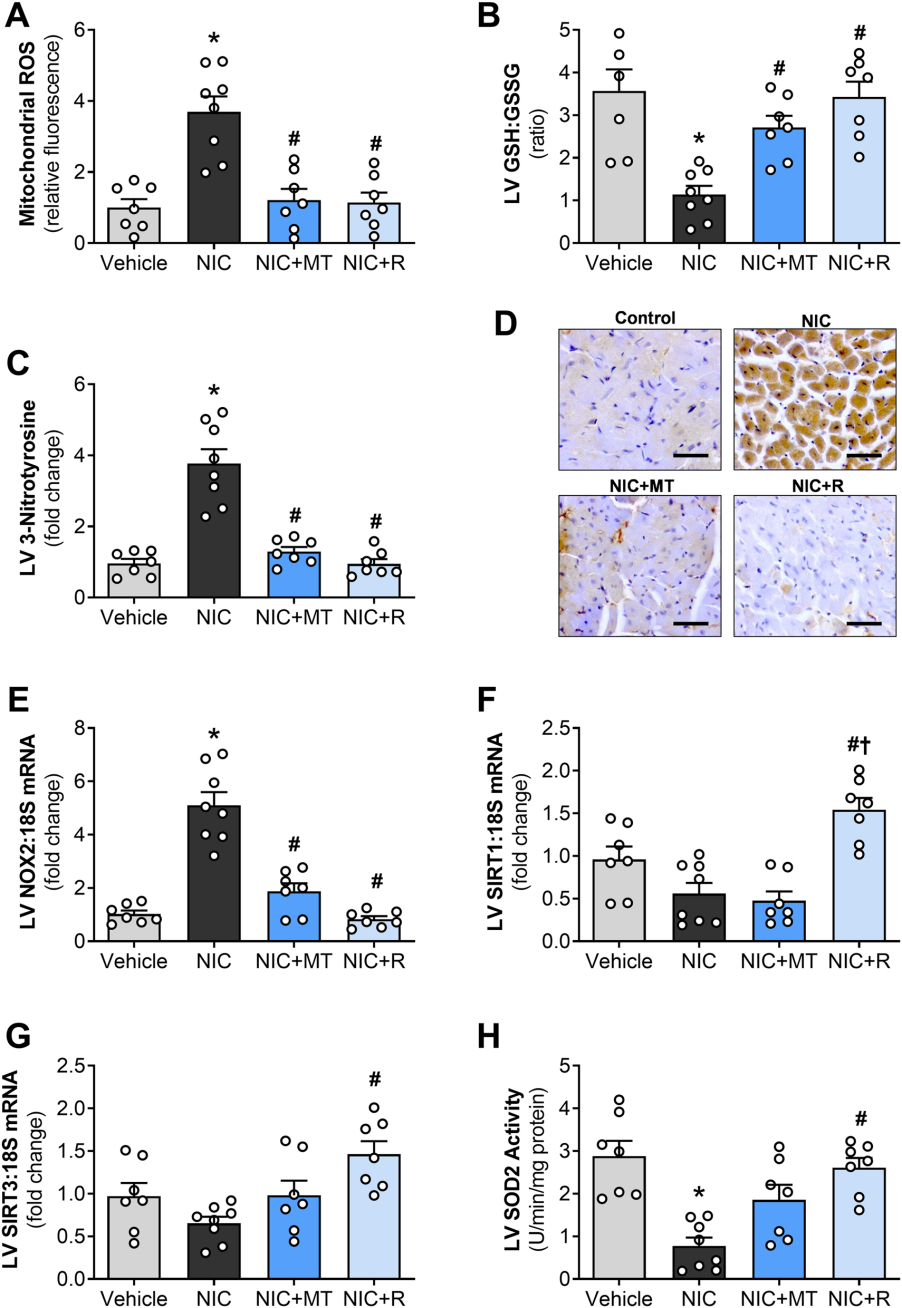
Impact of mitoTEMPO and resveratrol on nicotine-induced mitochondrial ROS-driven oxidative stress in rat hearts. (**A**) Mitochondrial ROS production, (**B**) GSH:GSSG ratio, (**C**) LV 3-nitrotyrosine content, (**D**) Representative images of LV sections immunostained with antibodies targeting 3-nitrotyrosine (scale bar = 50 µm), (**E**) LV gene expression of NOX2 subunit of NADPH oxidase, (**F**) LV gene expression of SIRT1, (**G**) LV gene expression of SIRT3 and (**H**) LV SOD2 activity. All values are given as mean ± SEM for *n* = 7–8/group; *p < 0.05 versus vehicle group; #*p* < 0.05 versus NIC group and †*p* < 0.05 versus NIC + MT group using one-way ANOVA with Tukey post hoc.

Prolonged nicotine administration for 28 days significantly increased mitochondrial ROS levels in rat hearts relative to the vehicle controls in this study (*p* < 0.05; Fig. 2A). Increased mitochondrial ROS emission was accompanied by considerably lowered glutathione ratio (GSH:GSSG) and markedly enhanced level of 3-nitrotyrosine in LV sections from nicotine-administered rats indicative of nicotine-induced oxidative stress in rat hearts (*p* < 0.05; Fig. 2B,C). Gene expression analysis revealed that nicotine administration significantly increased LV expression of Nox2 (*p* < 0.05 vs. controls; Fig. 2E). Compared to the nicotine-alone group, administration of mitoTEMPO and resveratrol both significantly blunted nicotine-induced increase in mitochondrial ROS emission after 28 days (*p* < 0.05; Fig. 2A). Both mitoTEMPO and resveratrol also preserved the myocardial GSH:GSSG ratio and significantly blunted nicotine-induced 3-nitrotyrosine level in rat hearts compared to the nicotine-alone group (*p* < 0.05; Fig. 2B–D). MitoTEMPO and resveratrol administration also significantly lowered LV expression of Nox2 compared to the nicotine-alone group (*p* < 0.05; Fig. 2E).

Gene expression analysis of the rat hearts also revealed that resveratrol administration alone and not mitoTEMPO significantly up-regulated LV expression of sirtuin isoforms, SIRT1 and SIRT3, as compared to the nicotine-alone group (*p* < 0.05 for both; Fig. 2F,G). Although nicotine administration tended to reduce SIRT1 gene expression (*p* = 0.08 vs. vehicle controls; Fig. 2F), nicotine administration had no significant effect on SIRT3 gene expression in this study (Fig. 2G). LV gene expression of the sirtuin-regulated endogenous antioxidant enzyme SOD2 was unaffected by all the treatments in this study. However, there was a non-significant trend for up-regulation in the resveratrol-administered group relative to the vehicle controls (Supplementary Figure S2). Nonetheless, nicotine administration significantly lowered SOD2 enzymatic activity in rat hearts after 28 days (*p* < 0.05 vs. vehicle controls, Fig. 2H). Resveratrol administration alone markedly increased the SOD2 enzymatic activity in rat hearts compared to the nicotine-alone group (*p* < 0.05; Fig. 2H).

### MitoTEMPO and resveratrol limited nicotine-induced cardiac remodeling in rats

**Figure 3:**
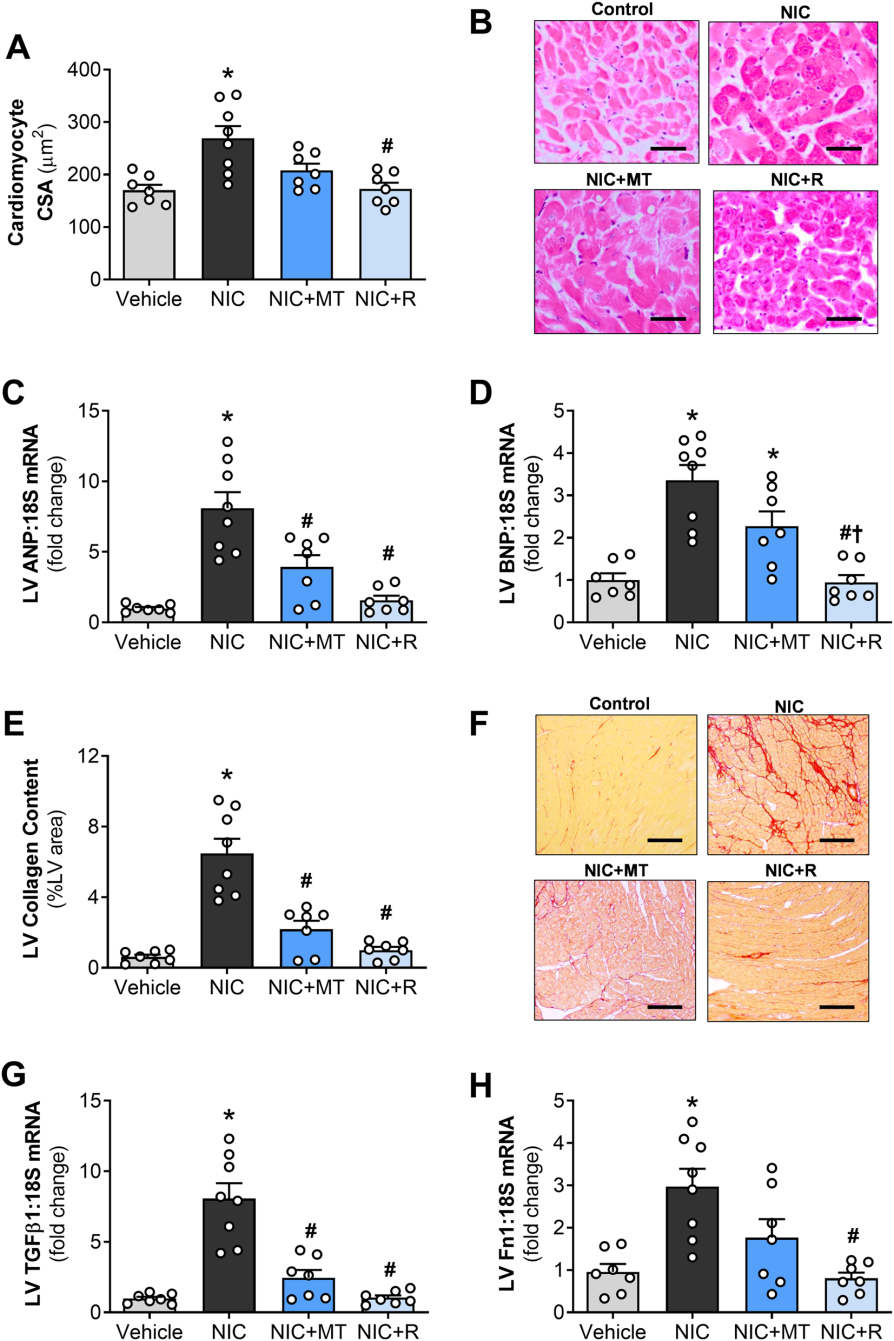
Impact of mitoTEMPO and resveratrol on nicotine-induced cardiac remodeling in rats. (**A**) Quantitative analysis of cardiomyocyte cross-sectional area (CSA) in LV tissues, (**B**) Representative images of H&E staining (Scale bar = 50 µm), (**C**) LV gene expression of ANP, (**D**) LV gene expression of BNP, (**E**) Quantitative analysis of collagen content in LV tissues, (**F**) Representative images of picrosirius-red staining (Scale bar = 200 µm) where collagen stains red in color, (**G**) LV gene expression of TGFβ1 and (**H**) LV gene expression of fibronectin. All values are given as mean ± SEM for *n* = 7–8/group; *p < 0.05 versus vehicle group; #*p* < 0.05 versus NIC group and †*p* < 0.05 versus NIC + MT group using one-way ANOVA with Tukey post hoc.

On quantitative analysis of cardiomyocyte size in H&E-stained LV sections, nicotine administration significantly increased cardiomyocyte cross-sectional area compared to the vehicle controls (*p* < 0.05; Fig. 3A). Our gene expression analysis also showed that LV expression of natriuretic peptides, ANP and BNP was significantly up-regulated in nicotine-administered rats compared to the vehicle controls (*p* < 0.05; Fig. 3C,D). Together with the increased LV weight and LV:TL ratio as reported in Table 1, these results indicate LV hypertrophy in nicotine-administered rats. MitoTEMPO administration failed to significantly attenuate the nicotine-induced increase in cardiomyocyte cross-sectional area in rat hearts after 28 days (Fig. 3A); however, it significantly normalized LV gene expression of ANP compared to the nicotine-alone group (*p* < 0.05; Fig. 3C). MitoTEMPO also showed a non-significant trend for reducing BNP gene expression compared to the nicotine-alone group (Fig. 3D). In contrast, resveratrol administration significantly attenuated the nicotine-induced increase in cardiomyocyte cross-sectional area and LV gene expression of both ANP and BNP compared to the nicotine-alone group (all *p* < 0.05; Fig. 3).

Evidence of pathological fibrosis was also observed in LV sections collected from nicotine-administered rats. On quantitative analysis of LV collagen density in picrosirius red-stained LV sections, nicotine administration significantly increased LV collagen deposition in rat hearts compared to the vehicle controls (*p* < 0.05; Fig. 3E). LV gene expression of fibrosis markers, TGFβ1 and Fn1, were both significantly up-regulated in nicotine-administered rats by 8.1-fold and 3.0-fold vs. vehicle controls, respectively (*p* < 0.05; Fig. 3G, H). MitoTEMPO administration significantly attenuated the nicotine-induced increase in LV collagen deposition and TGFβ1 upregulation compared to the nicotine-alone group (*p* < 0.05; Fig. 3G, H). Although there was a trend for reduced Fn1 expression in mitoTEMPO-administered rats compared to the nicotine-alone group, no statistical significance was attained. Conversely, resveratrol administration significantly attenuated nicotine-induced increases in all three markers compared to the nicotine-alone group (all *p* < 0.05; Fig. 3).

### MitoTEMPO and resveratrol modulated nicotine-induced cardiac inflammation in rats

**Figure 4:**
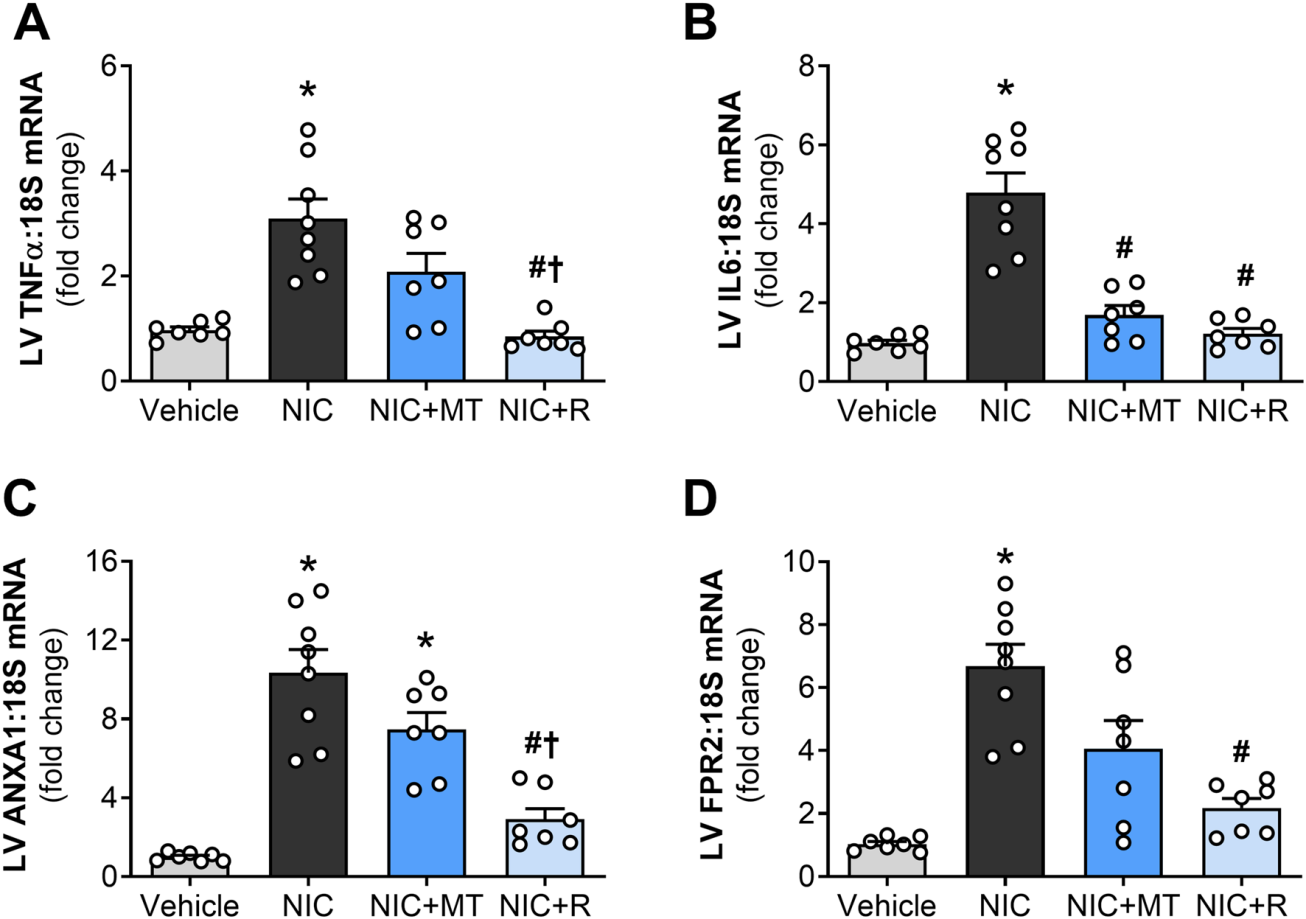
Impact of mitoTEMPO and resveratrol on inflammation in nicotine-administered rats. (**A**) LV gene expression of TNFα, (**B**) LV gene expression of IL-6, (**C**) LV gene expression of ANXA1, and (**D**) LV gene expression of FPR2. All values are given as mean ± SEM for *n* = 7–8/group; *p < 0.05 versus vehicle group, #*p* < 0.05 versus NIC group, †*p* < 0.05 versus NIC + MT group or NS (no statistical significance) using one-way ANOVA with Tukey post hoc.

LV gene expressions of pro-inflammatory cytokines, TNFα and IL-6, were significantly elevated in nicotine-administered rats compared to the vehicle controls (*p* < 0.05; Fig. 4A,B), indicative of cardiac inflammation. Although mitoTEMPO administration significantly reduced LV gene expression of TNFα (*p* < 0.05; Fig. 4A), it only tended to reduce LV gene expression of IL-6 in a non-statistically significant manner as compared to the nicotine-alone group (*p* = 0.08; Fig. 4B). Conversely, resveratrol administration significantly blunted nicotine-induced increases in LV expression of both TNFα and IL-6 as compared to the nicotine-alone group (*p* < 0.05; Fig. 4A,B). LV gene expression of the anti-inflammatory cytokine IL-10 was unaffected by all treatments relative to the vehicle controls or nicotine-alone group in this study (Supplementary Figure S5). Nonetheless, LV gene expression of the glucocorticoid-regulated anti-inflammatory molecule, ANXA1, and its receptor, FPR2, were both significantly up-regulated by nicotine administration as compared to the vehicle controls (*p* < 0.05 for both; Fig. 4C,D). Resveratrol significantly blunted nicotine-induced up-regulation of ANXA1 and FPR2 expression in this study (Fig. 4C,D). Although mitoTEMPO showed a trend for reduction in ANXA1 and FPR2, no statistical significance was attained.

### MitoTEMPO and resveratrol attenuated nicotine-induced cardiac dysfunction in rats

Cardiac function was determined ex vivo using Langendorff-perfused isolated rat hearts from all experimental groups (Table 2). Markers of LV function (LVSP, LVDP, LV + dP/dt, and LV − dP/dt) were all significantly reduced in nicotine-administered rats, as compared to the vehicle controls (*p* < 0.05; Table 2), indicative of cardiac dysfunction. Nicotine administration also markedly impaired baseline coronary flow rate in these hearts compared to the vehicle controls (*p* < 0.05; Table 2). However, mitoTEMPO and resveratrol significantly attenuated nicotine-induced reduction in LVSP, LVDP, LV + dP/dt, LV − dP/dt, and coronary flow rate (*p* < 0.05; Table 2), thus preventing nicotine-induced cardiac dysfunction in this study. Baseline LVEDP was unaffected by nicotine, mitoTEMPO and resveratrol (Table 2).

**Table 2:** End-point analysis of body weight, organ weight, and plasma cotinine in rats.

Parameters	Vehicle (n = 6)	NIC (n = 8)	NIC + MT (n = 8)	NIC + R (n = 7)
LVSP (mmHg)	78.2 ± 6.1	46.2 ± 7.2*	70.1 ± 6.2^#^	79.2 ± 4.9^#^
LVEDP (mmHg)	2.0 ± 0.8	2.2 ± 0.7	1.9 ± 0.7	2.1 ± 0.9
LVDP (mmHg)	75.1 ± 5.2	43.4 ± 6.2*	68.3 ± 7.4^#^	76.4 ± 9.3^#^
LV + dP/dt (mmHg/s)	2633.2 ± 112.1	1645.4 ± 134.7*	2349.6 ± 133.2^#^	2321.4 ± 149.7^#^
LV – dP/dt (mmHg/s)	1831.8 ± 134.2	1012.9 ± 56.3*	1723.2 ± 91.7^#^	1909.5 ± 124.2^#^
Coronary flow (mL/min)	12.2 ± 1.2	6.1 ± 0.9*	11.0 ± 1.1^#^	`12.9 ± 1.5^#^
Heart rate (bpm)	261.8 ± 35.3	220.3 ± 33.8	255.7 ± 31.8	270.8 ± 43.1

All values are given as mean ± SEM for *n* = 6–8/group.

*LVDP* left ventricular developed pressure, *LVEDP* left ventricular end-diastolic pressure, *LV + dP/dt* maximal rate of left ventricular contraction, *LV – dP/dt* maximal rate of left ventricular relaxation, *LVSP* left ventricular systolic pressure, *NIC* nicotine, *NIC + MT* nicotine + mitoTEMPO, *NIC + R* nicotine + resveratrol.

*p < 0.05 versus vehicle group.

^#^*p* < 0.05 versus NIC group using one-way ANOVA with Tukey post-hoc test.

### MitoTEMPO and resveratrol attenuated nicotine-associated aggravation of the myocardial ischemia–reperfusion injury in rat hearts ex vivo

**Figure 5:**
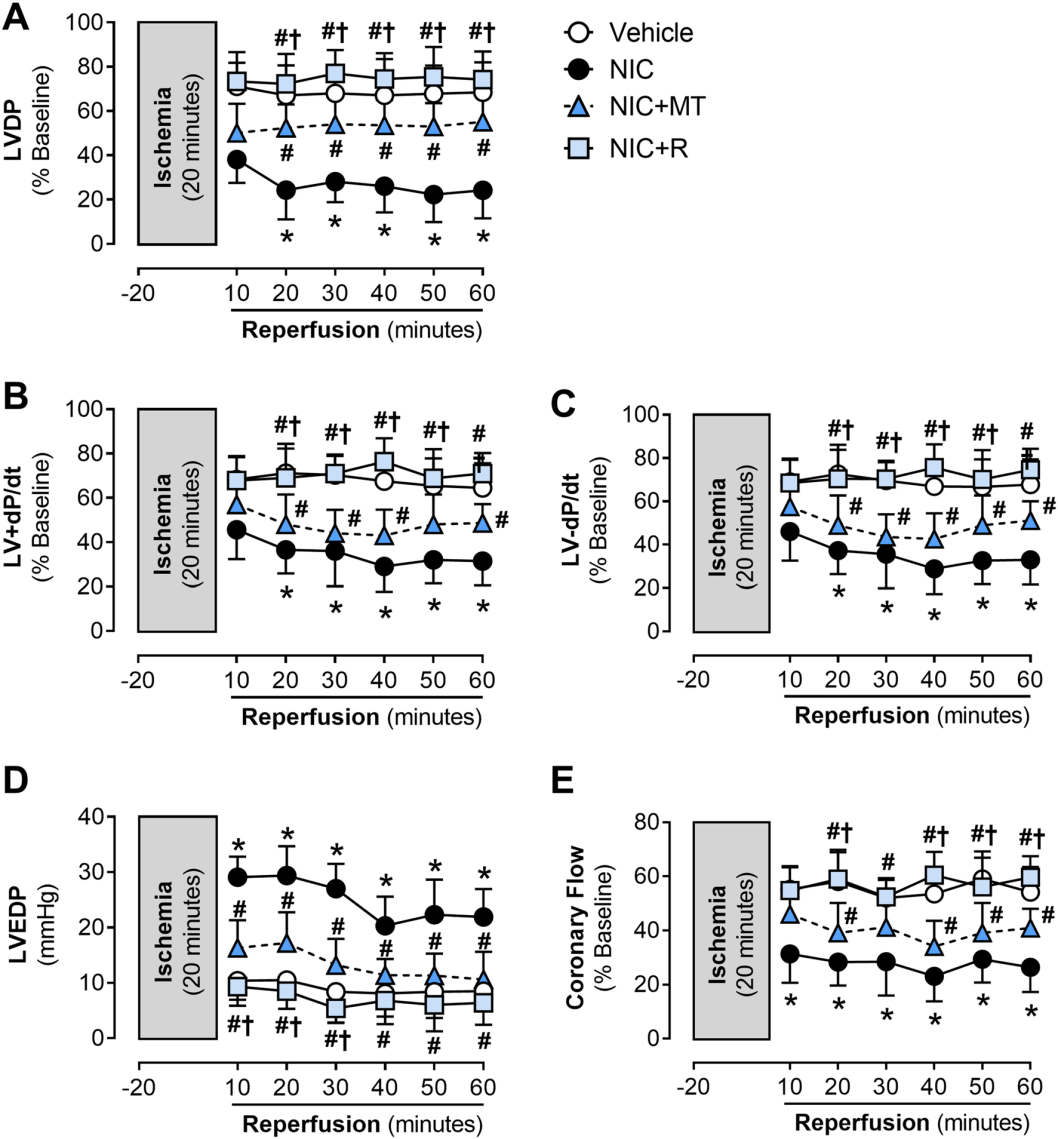
Time course changes in post-ischemic recovery of (**A**) LVDP, (**B**) LV + dP/dt, (**C**) LV − dP/dt, (**D**) LVEDP, and (**E**) coronary flow in Langendorff-perfused rat hearts isolated from the vehicle, NIC, NIC + MT, and NIC + R. All values are given as mean ± SEM for *n* = 6–8/group; *p < 0.05 versus vehicle group; #*p* < 0.05 versus NIC group and †*p* < 0.05 versus NIC + MT group using two-way ANOVA with repeated measurement.

Figure 5 shows the time course changes in post-ischemic recovery of LV function and coronary flow in Langendorff-perfused rat hearts isolated from all experimental groups. Compared to the vehicle controls, nicotine administration significantly impaired percent recovery of LVDP, LV + dP/dt, and LV − dP/dt (*p* < 0.05; Fig. 5A–C). Administration of mitoTEMPO and resveratrol significantly improved post-ischemic recovery of LV function, as shown by improvement across all three markers evident from the 10-min onset of reperfusion in this study (*p* < 0.05; Fig. 5A–C). Nicotine administration also induced a significant increase in LVEDP in isolated rat hearts during reperfusion compared to the vehicle controls (*p* < 0.05; Fig. 5D). An increase in LVEDP after ischemia–reperfusion is an indicator of impaired LV relaxation, which further suggests nicotine-associated aggravation of the myocardial ischemia–reperfusion injury. MitoTEMPO and resveratrol administration significantly reduced LVEDP elevation in the isolated rat hearts (Fig. 5D). Nicotine-induced reduction in coronary flow was further exacerbated following ischemia–reperfusion compared to the vehicle controls. This condition was also attenuated by both mitoTEMPO and resveratrol administration (*p* < 0.05 for both; Fig. 5E). Improvement in coronary function was evident from the 10-min onset of reperfusion for both mitoTEMPO and resveratrol-administered groups. There was a significant difference between both groups at each time point (Fig. 5E).

**Figure 6:**
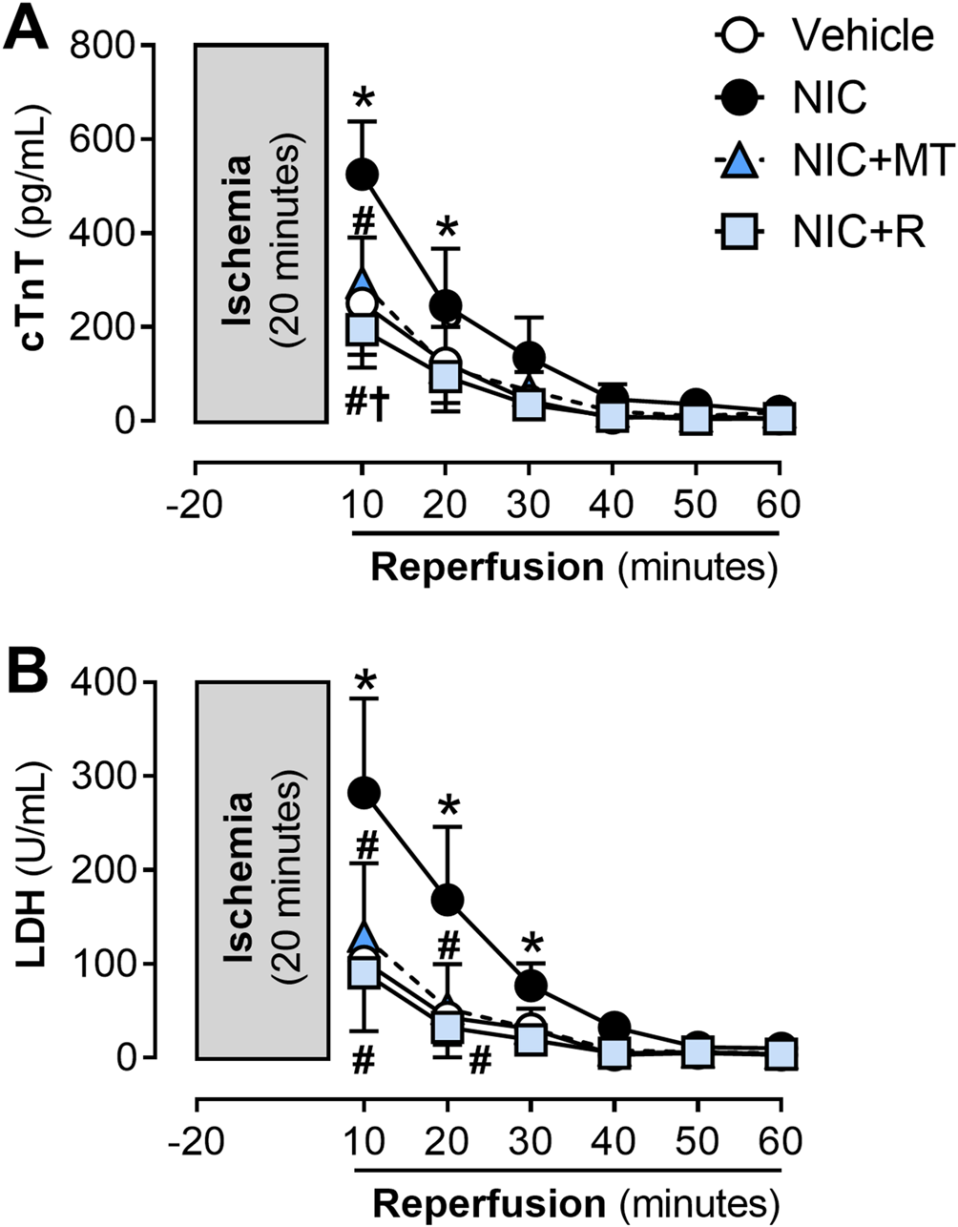
Time course for post-ischemic release of cardiac injury markers, (**A**) cTnT and (**B**) LDH in coronary effluent from Langendorff-perfused rat hearts isolated from the vehicle, NIC, NIC + MT, and NIC + R. All values are given as mean ± SEM for *n* = 6–8/group; *p < 0.05 versus vehicle group; #*p* < 0.05 versus NIC group and †*p* < 0.05 versus NIC + MT group using two-way ANOVA with repeated measurement.

Analysis of cardiac injury markers released in coronary effluent throughout reperfusion also revealed that nicotine administration markedly increased LDH and cardiac TnT release from Langendorff-perfused rat hearts at the onset of reperfusion (*p* < 0.05; Fig. 6A,B). MitoTEMPO and resveratrol significantly attenuated nicotine-induced increases in LDH release and cardiac TnT release (*p* < 0.05; Fig. 6A,B).

## Discussion

In this study, we have demonstrated for the first time that targeting mitochondrial ROS using mitochondria-targeted SOD mimetic, mitoTEMPO, and the natural red wine constituent, resveratrol blunts and partially attenuates pathogenesis of nicotine-induced cardiac remodeling, dysfunction, and associated aggravation of the myocardial ischemia–reperfusion injury. We showed that mitoTEMPO and resveratrol attenuated nicotine-induced oxidative stress, inflammation, and cardiac remodeling such as hypertrophy and fibrosis in a rat model. MitoTEMPO and resveratrol administration also caused partial attenuation of nicotine-induced cardiac dysfunction and associated aggravation of ischemia–reperfusion injury as determined in isolated Langendorff-perfused rat hearts.

Numerous studies have identified mitochondrial ROS as the primary contributor to oxidative stress-driven cardiovascular diseases such as hypertension, diabetic cardiomyopathy, and ischemia–reperfusion injury^14,15,33^. Unregulated mitochondrial ROS production interferes with energy production in mitochondria and perturbs calcium signaling, increases apoptosis, and mediates pro-hypertrophic and fibrotic signaling pathways in the heart^15,16,33^. In this study, nicotine administration for 28 days significantly enhanced mitochondrial ROS emission in rat hearts, accompanied by a decrease in GSH:GSSG ratio, an increase in 3-nitrotyrosine level and an up-regulation of Nox2 gene expression. Both mitoTEMPO and resveratrol significantly blunted nicotine-induced mitochondrial ROS production, normalized GSH:GSSG ratio, reduced 3-nitrotyrosine level, and down-regulated Nox2 gene expression. Consistent with our hypothesis, this shows that both mitoTEMPO and resveratrol targeted mitochondrial ROS production in nicotine-administered rats and prevented subsequent oxidative stress that is implicated in the development of cardiac hypertrophy, fibrosis, and dysfunction.

In this study, we also studied gene expression of sirtuins (SIRT1 and SIRT3) and sirtuin-regulated mitochondrial antioxidant (SOD2) in rat hearts to determine whether protective actions of resveratrol is associated with sirtuin-mediated activation of SOD2 in mitochondria. Indeed, resveratrol induces activation of multiple sirtuins isoforms such as SIRT1 and SIRT3. Sirtuins are highly conserved anti-aging molecules that are frequently implicated as key protective molecules responsible for the benefits of red wine consumption and caloric restriction^34^. Sirtuin activation has been shown to target mitochondrial oxidative stress via up-regulation of the SOD2 enzyme^35^. SOD2 selectively scavenges mitochondrial ROS and improves cardiac function when overexpressed^36^. Our gene expression analysis in this study revealed that nicotine administration for 28 days tended to reduce SIRT1 gene expression, consistent with a previous report that suggested nicotine reduces SIRT1 expression in mice^37^. Nicotine administration, however, had no significant effect on the SIRT3 gene expression. Resveratrol alone and not mitoTEMPO significantly up-regulated SIRT1 and SIRT3 gene expression compared to the nicotine-administered rats. We further noted that SOD2 gene expression was not affected by all treatments in this study (Supplementary Figure S2), although nicotine significantly reduced SOD2 enzyme activity (Fig. 2H). However, resveratrol significantly increased SOD2 enzyme activity in rat hearts compared to the nicotine-alone group (Fig. 2H). Our observation shows that sirtuin-mediated activation of SOD2 may contribute in part to the reduced mitochondrial ROS production in resveratrol-administered animals in our study. MitoTEMPO had no significant effect on both gene expression and activity of SOD2, thus reduced mitochondrial ROS production seen in mitoTEMPO-administered animals is likely mediated by direct ROS scavenging action of mitoTEMPO. The fact that mitoTEMPO and resveratrol both attenuated mitochondrial ROS emission but only resveratrol improved SIRT3 expression indicates that targeting mitochondrial oxidative stress alone is in part a primary therapeutic target for nicotine-induced cardiac dysfunction in this study. It should also be noted that sirtuins are also implicated in activating metabolic regulatory proteins such as PGC1α^38^. Therefore, future studies should examine whether cardiac metabolic remodeling plays a role in nicotine-induced cardiac dysfunction to identify whether sirtuin-mediated regulation of cardiac metabolism underlies resveratrol-induced protection in nicotine-administered animals.

Maladaptive LV hypertrophy is an independent predictor for heart failure and is associated with an increased risk for sudden cardiac death^39^. In this study, nicotine-induced LV hypertrophy was evident from increased LV weight, LV:TL ratio, cardiomyocyte cross-sectional area, and gene expression of natriuretic peptides (ANP and BNP), consistent with our previous study^9^. Nicotine-induced LV hypertrophy is likely, in part, due to the increase in blood pressure and oxidative stress. MitoTEMPO did not significantly reduce nicotine-induced cardiomyocyte enlargement measured from H&E-stained sections, although it showed a tendency to attenuate BNP up-regulation and significantly reduced ANP gene expression. MitoTEMPO also prevented the nicotine-induced increase in LV weight and LV:TL ratio suggesting it does have the potential to avert LV hypertrophy, albeit with lower efficiency in preventing cardiomyocyte enlargement at the cellular level. In contrast, resveratrol administration significantly attenuated the nicotine-induced increase in all cardiac hypertrophy markers, including cardiomyocyte cross-sectional area, ANP, BNP, LV weight, and LV:TL ratio. Resveratrol was likely more effective in preventing nicotine-induced cardiac hypertrophy due to the combined action of ROS scavenging, blood pressure-lowering, and sirtuin activation.

Interstitial fibrosis, which often occurs together with LV hypertrophy, was also noted in nicotine-administered rats in this study, as shown by increased collagen deposition on picrosirius red staining and fibronectin gene expression. Several studies have suggested that TGFβ signaling is involved in nicotine-induced fibrogenesis^12,40^; therefore, we investigated gene expression of TGFβ1 in our rat model. TGFβ1 modulates proliferation, migration, and differentiation of fibroblasts that produce collagen and alter the cardiac extracellular matrix^41^. Consistent with our previous report^9^, we noted increased TGFβ1 gene expression in nicotine-administered rats that may mediate the cardiac fibrosis observed among these rats. Nonetheless, both mitoTEMPO and resveratrol significantly prevented nicotine-induced cardiac fibrosis by inhibiting TGFβ1 up-regulation and reducing collagen deposition in this study. Resveratrol also remarkably reduced fibronectin gene expression, while mitoTEMPO showed a non-significant tendency to reduce its expression. ROS regulates fibrosis in the heart via various mechanisms, including TGFβ signaling, fibroblast apoptosis, and increased collagen degradation^42^. Thus, our data highlights that mitoTEMPO and resveratrol both prevented nicotine-induced cardiac fibrosis, at least partly via the reduction in mitochondrial ROS production.

Cardiac inflammation was also evident in nicotine-administered rats in this study. Nicotine administration markedly increased LV gene expression of pro-inflammatory cytokines such as TNFα and IL-6, similar to our previous study^9^, and these may be responsible for nicotine-induced cardiac remodeling and dysfunction. Administration of mitoTEMPO and resveratrol significantly ameliorated nicotine-induced upregulation in TNFα and IL-6 gene expression. MitoTEMPO (0.6 mg/kg) was also previously shown to abolish the expression of inflammatory cytokines in an animal model of hypertension, primarily via reduction of mitochondrial oxidative stress^43^. Mitochondrial ROS is critical for activation of the inflammasome and inflammatory signaling^44^; therefore, it is possible that ROS scavenging effect of mitoTEMPO prevented macrophage activation and suppress the production of pro-inflammatory cytokines in nicotine-administered rats. Resveratrol elicits anti-inflammatory activity in various in vitro and in vivo inflammatory disease models independent of reduced oxidative stress^45,46^. Therefore, resveratrol may have inhibited nicotine-induced cardiac inflammation also in part via direct inhibition of macrophage infiltration, expression of NF-κB expression, and suppression of inflammatory cytokines as previously reported^46^.

In this study, nicotine administration for 28 days did not reduce LV gene expression of IL-10 (Supplemental Figure S5); however, nicotine significantly up-regulated LV gene expression of ANX-A1 and its receptor, FPR2, that modulates IL-10 production. ANX-A1 is an endogenous glucocorticoid-regulated peptide whose expression is necessary for the resolution of inflammation^47^. Activation of ANX-A1/FPR2 signaling limits inflammation via accelerated neutrophil clearance and production of anti-inflammatory cytokines such as IL-10^48^. ANX-A1 up-regulation in nicotine-administered rats is likely a compensatory response towards nicotine-induced inflammation. MitoTEMPO administration failed to significantly blunt nicotine-induced up-regulation of ANXA1 and FPR2 in this study, although there was a trend for reduction in ANXA1 and FPR2 expression. This was surprising given that we previously reported that exogenous administration of SOD mimetic, Tempol attenuated renal ANX-A1 expression and inflammation in concert with blunted oxidative stress in a rat model of type I diabetes^49^. One possible explanation for this observation is that extramitochondrial ROS may contribute primarily to the regulation of ANX-A1 and Tempol, unlike mitoTEMPO, was capable of successfully scavenging extramitochondial ROS that regulated ANX-A1 expression. Future studies are warranted to test this hypothesis. Resveratrol administration conversely normalized ANXA1 and FPR2 gene expression in this study. Considering that both mitoTEMPO and resveratrol reduce nicotine-induced inflammation, it is likely that there was no need for compensatory up-regulation of ANXA1 and FPR2 expression to promote resolution of inflammation.

Consistent with our previous report^9,10^, cardiac dysfunction was evident in Langendorff-perfused rat hearts isolated from nicotine-administered rats in this study. Markers of LV contractile function (LVDP, LV + dP/dt, and LV − dP/dt) and coronary flow rate were all significantly impaired in the nicotine-alone group, indicative of contractile dysfunction and impaired coronary flow. Recovery of LVDP, LV + dP/dt, and LV − dP/dt further deteriorated during reperfusion. The coronary flow rate was also similarly impaired. We also noted that LVEDP in the nicotine group gradually increased throughout reperfusion, indicating impaired ventricular relaxation due to ischemia–reperfusion injury^9,10^. Several mechanisms may have contributed to nicotine-induced cardiac dysfunction and aggravation of the ischemia–reperfusion damage, including excess mitochondrial ROS emission, oxidative stress, cardiac remodeling such as hypertrophy and fibrosis, as well as increased cardiomyocyte injury. Indeed, hypertrophied and remodeled hearts are more vulnerable to acute ischemic insults^50^. Extensive collagen deposition may also stiffen the myocardium and impair ventricular relaxation. In this study, mitoTEMPO and resveratrol mitigated nicotine-induced cardiac dysfunction and aggravation of the myocardial ischemia–reperfusion injury. This could be attributed primarily to their ability to reduce mitochondrial ROS emission, oxidative stress, inflammation, cardiac hypertrophy, and fibrosis. However, it is noteworthy that resveratrol demonstrated more effectiveness in attenuating nicotine-induced cardiac dysfunction and aggravation of ischemia–reperfusion injury compared to mitoTEMPO, as shown by recovery of LV function throughout reperfusion.

Hypertension is independently associated with increased myocardial remodeling and heart failure incidence^51^. This study observed that nicotine consistently increased systolic blood pressure in nicotine-administered rats over 4 weeks. It is highly likely that increased blood pressure may also contribute in part to the cardiac remodeling and dysfunction observed in nicotine-administered animals. Blood pressure-lowering activity of mitoTEMPO and resveratrol that was previously documented^14,52^ could have contributed in part to their protection against nicotine-induced cardiac remodeling and dysfunction in this study. However, it should be noted that multiple studies have reported that mitoTEMPO and resveratrol effectively attenuate cardiac oxidative stress, hypertrophy, fibrosis, and dysfunction independent of blood pressure-lowering effect^15,22^.

### Study limitation

In this study, cardiac function was determined ex vivo using the Langendorff perfusion technique, which does not wholly replicate in vivo analysis of cardiac function. This setup fails to account for the influence of circulating inflammatory cells, hormones, and chemokines. In vivo cardiac function analysis using echocardiography is the most used method for routine detection of cardiac dysfunction in clinical settings. Therefore, future studies should examine the impact of nicotine administration, alone or in combination with mitochondria-targeted antioxidants, on echocardiographic measures of cardiac function in vivo. Further study should be warranted to look into pressure–volume loop analysis, which could provide better insights on understanding whether changes in LVEDP can influence the systolic function of rat hearts in the treatment groups. Several fold changes in mRNA do not necessarily imply the corresponding change in the protein levels. In this study, we also noted that mitoTEMPO and resveratrol reduced blood pressure:therefore, the cardioprotective effects of mitoTEMPO and resveratrol could be direct and indirect (via reduction of hypertension).

## Conclusion

This study demonstrated that targeting mitochondrial ROS-driven oxidative stress with mitoTEMPOor resveratrol attenuates partially nicotine-induced cardiac remodeling, dysfunction, and associated aggravation of myocardial ischemia–reperfusion injury in a rat model. MitoTEMPO and resveratrol also showed attenuation of nicotine-induced hypertension after 28 days. Increased mitochondrial ROS emission, secondary to nicotine administration, may precede and mediate inflammation, LV hypertrophy, fibrosis, and contractile dysfunction, which render the hearts more vulnerable to ischemia–reperfusion injury in active and passive smokers. Evidence from this study also supports the hypothesis that the development of novel mitochondria-targeted antioxidants may serve as more efficient therapeutic agents in cardiovascular diseases.

## Supplementary Information

Supplementary Information.

## Data Availability

Data are available from the authors (AR and SZ) upon reasonable request.
