# Supplementary material for: Targeting mitochondrial reactive oxygen species-mediated oxidative stress attenuates nicotine-induced cardiac remodeling and dysfunction
Source: Sci Rep. 2021 Jul 5;11:13845. doi: 10.1038/s41598-021-93234-4 (PMC8257608; doi:10.1038/s41598-021-93234-4)
Supplement: Supplementary file 1 — Supplementary Information. [file 41598_2021_93234_MOESM1_ESM.docx]

*Supplementary File*

**Targeting mitochondrial reactive oxygen species-mediated oxidative stress attenuates nicotine-induced cardiac remodeling and dysfunction**

Anand Ramalingam^1^, Siti Balkis Budin^2^, Norsyahida Mohd. Fauzi^3^, Rebecca H. Ritchie^4,5^, Satirah Zainalabidin^1,^*

1. Programme of Biomedical Science, Centre of Toxicology and Health Risk Studies, Faculty of Health Sciences, Universiti Kebangsaan Malaysia, Kuala Lumpur, Malaysia
2. Programme of Biomedical Science, Centre of Diagnostic, Therapeutic and Investigative Studies, Faculty of Health Sciences, Universiti Kebangsaan Malaysia, Kuala Lumpur, Malaysia
3. Drug and Herbal Research Centre, Faculty of Pharmacy, Universiti Kebangsaan Malaysia, Jalan Raja Muda Abdul Aziz, Kuala Lumpur, Malaysia
4. Heart Failure Pharmacology, Baker Heart and Diabetes Institute, Melbourne, VIC, Australia
5. Drug Discovery Biology, Monash Institute of Pharmaceutical Sciences, Monash University, Parkville, VIC, Australia

***Correspondence:** satirah@ukm.edu.my; Tel.: +603-92897684

**Supplementary Table 1.** Primer sequence for quantitative real time PCR analysis.

| **Gene Name** | **Primer Sequence (5’ to 3’)** | |
| --- | --- | --- |
| 18S | Forward | TTCGAGGCCCTGTAATTGGA |
|  | Reverse | GCAGCAACTTTAATATAGGCTATTGG |
| SIRT1 | Forward | TGCCATCATGAAGCCAGAGA |
|  | Reverse | CATCGCAGTCTCCAAGAAGC |
| SIRT3 | Forward | GGCGTTGTGAAACCTGACAT |
|  | Reverse | AGGACTCAGAGCAAAGGACC |
| SOD2 | Forward | ACAGGCCTTATTCCACTGCT |
|  | Reverse | CTACAAAACACCCACCACGG |
| ANP | Forward | GGAAGTCAACCCGTCTCAGA |
|  | Reverse | TGGGCTCCAATCCTGTCAAT |
| BNP | Forward | ACAAGAGAGAGCAGGACACC |
|  | Reverse | TCTGGAGACTGGCTAGGACT |
| TGFβ1 | Forward | CCTGCAAGACCATCGACATG |
|  | Reverse | TGTTGTACAAAGCGAGCACC |
| Fibronectin | Forward | GAAAGGCAACCAGCAGAGTC |
|  | Reverse | CTGGAGTCAAGCCAGACACA |
| Nox2 | Forward | CTGCCAGTGTGTCGGAATCT |
|  | Reverse | TGTGAATGGCCGTGTGAAGT |
| TNFα | Forward | ACACACGAGACGCTGAAGTA |
|  | Reverse | GGAACAGTCTGGGAAGCTCT |
| IL-6 | Forward | TCTCTCCGCAAGAGACTTCCA |
|  | Reverse | ATACTGGTCTGTTGTGGGTGG |
| IL-10 | Forward | CCTGCTCTTACTGGCTGGAG |
|  | Reverse | TGTCCAGCTGGTCCTTCTTT |
| ANXA1 | Forward | GCCCCTACCCTTCCTTCAAT |
|  | Reverse | GCCAAAACAACCTCCTCCAG |
| FPR2 | Forward | ATCTGGGTAGCTGGATTCCG |
|  | Reverse | GGATGCAGGACACAAATGCA |

**Supplementary Figures**


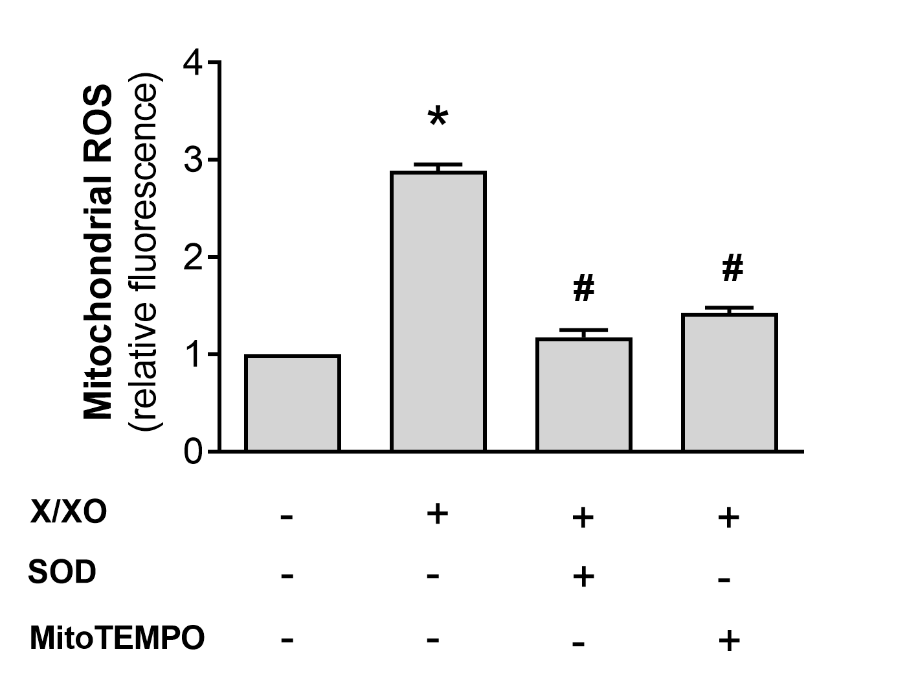


**Fig. S1.** Direct ROS scavenging by MitoTEMPO in isolated rat heart mitochondria. Rat heart mitochondria were incubated with xanthine and xanthine oxidase (X/XO) with or without mitoTEMPO or bovine superoxide dismutase (SOD) for 30 minutes and ROS production was monitored using MitoSOX assay. *p<0.05 vs. negative control and #p<0.05 vs. X/XO only control using one-way ANOVA with Tukey post-hoc for n=4 independent experiments.


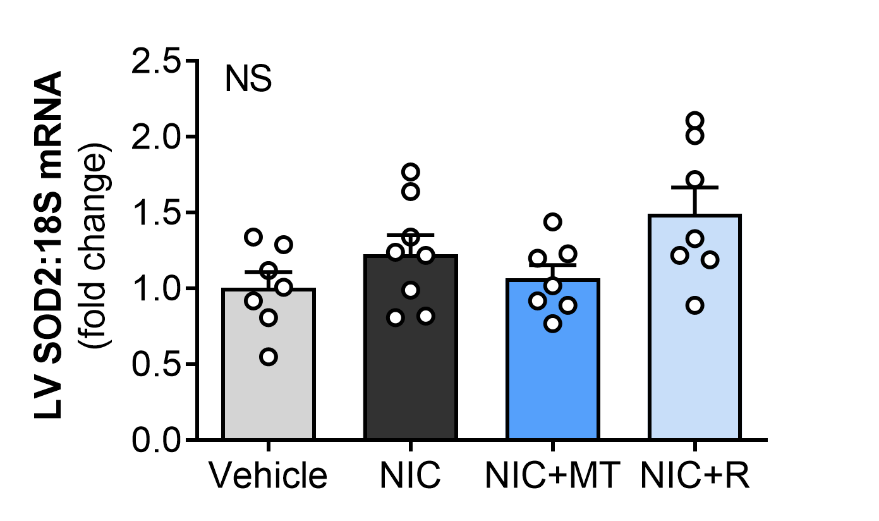


**Fig. S2.** Impact of mitoTEMPO and resveratrol on LV SOD2 gene expression in rats. All values are given as mean ± SEM for *n*=7-8/group; NS (no statistical significance) using one-way ANOVA with Tukey post-hoc.


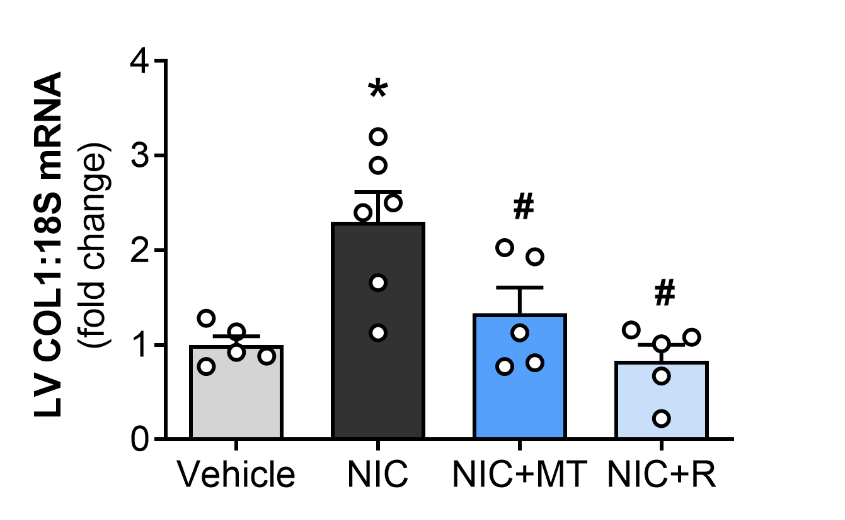


**Fig. S3.** Impact of mitoTEMPO and resveratrol on LV collagen I (COL1) gene expression in rats. All values are given as mean ± SEM for *n*=5-6/group; *p<0.05 vs. vehicle controls and #p<0.05 vs. NIC group using one-way ANOVA with Tukey post-hoc.


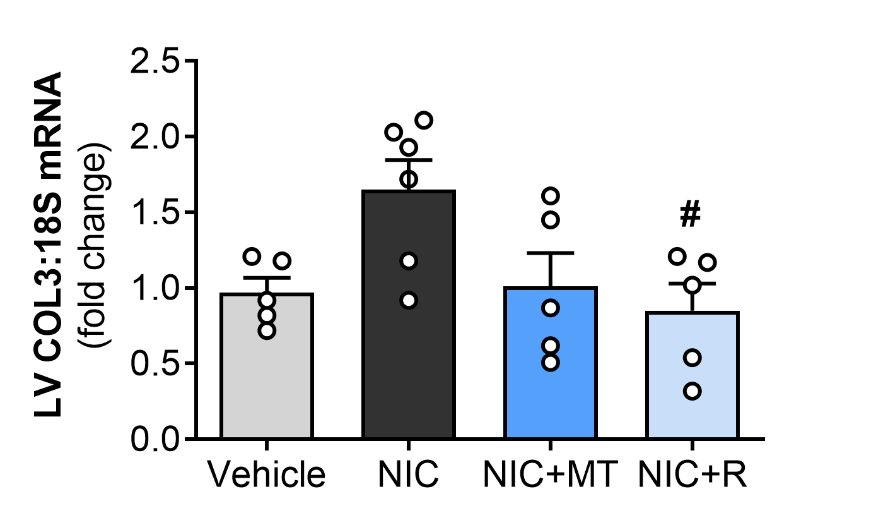


**Fig. S4.** Impact of mitoTEMPO and resveratrol on LV collagen III (COL3) gene expression in rats. All values are given as mean ± SEM for *n*=5-6/group; *p<0.05 vs. vehicle controls and #p<0.05 vs. NIC group using one-way ANOVA with Tukey post-hoc.


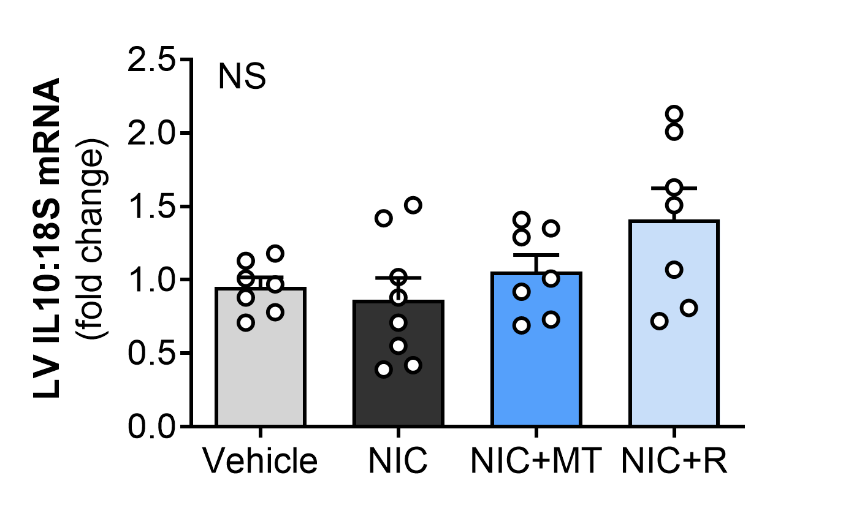


**Fig. S5.** Impact of mitoTEMPO and resveratrol on LV interleukin 10 (IL-10) gene expression in rats. All values are given as mean ± SEM for *n*=7-8/group; NS (no statistical significance) using one-way ANOVA with Tukey post-hoc.
